# Investigating Foot Morphology in Rock Climbing Mammals: Inspiration for Biomimetic Climbing Shoes

**DOI:** 10.3390/biomimetics8010008

**Published:** 2022-12-24

**Authors:** Stephen Spurrier, Tom Allen, Robyn A. Grant

**Affiliations:** Faculty of Science and Engineering, Manchester Metropolitan University, Manchester M1 5GD, UK

**Keywords:** shoes, footwear, sport, design, foot, feet, digit, pad, principal component analysis, shape, size

## Abstract

The sporting goods sector can serve as a proving ground for new technologies. We propose that climbing shoes are an excellent case study for showcasing a systematic approach to bio-inspired design. Foot adaptations to climbing have been described before in some animals and have even been incorporated into bio-inspired products. However, there has not yet been a systematic description of climbing adaptations in mammals, and especially in rock climbing species. We present a description of foot morphology in mammals and compare rock climbing species to those with other locomotion types. Our results show that rock climbing species in our sample had fewer digits and larger anterior pads than arboreal species. Rock climbing species often had hooves or, if they had foot pads, these were relatively smooth. These examples look a bit like current climbing shoe designs, perhaps suggesting convergent evolution. However, there was also variation, with rock climbing species having pads varying in shape, placement and texture. Much of this variation is likely to be dependent on the relatedness of species, with those that are more related having more similar feet. We suggest that incorporation of novel textures and compliant pads might be an interesting focus for future climbing shoe designs.

## 1. Introduction

Taking inspiration from millions of years of natural evolution, bio-inspired design is thought to have the potential to improve products [1,2]. Overall, two main approaches are taken to bio-inspired design. One of these approaches is to identify *a natural solution* and use it to inspire a new or improved product; whereas the other is to solve *a design problem* with inspiration from nature [1,2,3]. It can often be unclear how the biological innovation example itself is selected and there is little evidence in the literature to suggest that comprehensive and systematic biological example selection always occurs. Indeed, sometimes only one natural example is given, with limited justification as to why that one is chosen specifically.

The rapid uptake sporting goods sector makes an ideal test arena for new technologies and processes [4], such as bio-inspired design. Examples of sport and exercise products where bio-inspired design concepts have already been applied include swimsuits [5], watersports boards [6], backpacks [2], knee protectors [3] and other protective equipment [7,8,9]. There is certainly opportunity to continue to take inspiration from nature to bring improvements to other sporting goods.

Improvements in sporting goods are often gradual and incremental, with occasional step changes and innovations, which improves performance in the sport [10,11,12,13,14,15,16,17,18,19,20,21]. These equipment developments can be driven by various factors, including user preferences and trends, new materials and manufacturing processes, and innovative design techniques [10,13,21]. For example, rock climbing footwear has developed from adapted hiking boots to specialist tight-fitting shoes with stiff “grippy” rubber soles. Despite these developments, the few scientific publications on climbing shoes have focused on how an overly tight fit can cause discomfort and foot injuries [22,23,24], rather than how design influences performance. With many diverse examples of climbing animals, the climbing shoe is particularly well-suited for showcasing a comprehensive and systematic process for selecting natural examples for bio-inspired design. Following such a process should lead to a rich variety of bio-inspired design concepts and could lead to further developments of the climbing shoe.

Indeed, climbing is a prevalent locomotion type across animals and has led to various morphological adaptations of the foot. Overall, these adaptations can be split into two methods. One of these methods interlocks the foot with a surface, such as by having deformable pads, gripping paws or claws; whereas the other develops bonds between the foot and surface, such as by applying adhesion or suction [25]. These natural adaptations to aid climbing have already inspired engineering designs, including robots that climb (e.g., gecko [26,27]) and walk (e.g., goat [28]), and adhesive nanoparticle innovations (e.g., climbing plants [29]).

Animals that use adhesive pads for climbing vary in size from small mites to large geckos [30], but there are challenges with this approach as animals get even larger. For example, larger mammal and human feet attach less well to surfaces, due to their smaller surface-to-volume ratio, and because it becomes increasingly difficult to distribute load uniformly across large contact areas [30]. Therefore, interlocking mechanisms, like those observed in larger animals, especially mammals, might be better aligned to inspire a biomimetic human climbing shoe, rather than adhesive mechanisms, like those seen in smaller animals, such as mites and geckos [30]. There are certainly many foot shapes and structures in mammals, which are associated with climbing adaptations. For example, climbing primates and rodents both have long, moveable digits for grasping tree branches [25,31]. Climbing primates and marsupials both have reduced claws that do not impede grip, while, conversely, sloths have long claws for hanging from tree branches [32]. Rodents and small primates have many, textured, deformable foot pads [31,32]. The convex shape, compliant nature and texture of these foot pads increase surface contact area and improves grip during climbing [31]. These small pads also enable precise paw movements during climbing, as well as during object manipulation [32]. Conversely, larger primates tend to have fewer and larger foot pads, which helps to provide a uniform contact area for tree climbing [25].

While studies have described foot adaptations in detail in primates, and in some depth in rodents, and marsupials [31,33,34], there has been no formal, systematic description of climbing adaptations across more diverse mammalian groups. Furthermore, studies have tended to focus on arboreality (tree climbing) [25,35], due to the first mammals being arboreal [36], and most extant climbing mammals still being arboreal, rather than rock climbers [25]. However, rock climbing is present across mammalian species (including in species of goat, sheep, deer, pika, and tenrec, among others), despite it being relatively understudied. Abad et al. [28] briefly described the morphology of goat hooves, which contain a shell of hard keratin and a compliant, textured pad to increase friction and stability on uneven ground, although they only investigated one species (the Ecuadorian mountain goat). As such, there is scope to explore and describe foot adaptations in a range of mammals, and especially with a focus on adaptations to rock climbing [35]. The aim of this study, therefore, is to describe the foot morphology of mammals and explore its association with Locomotion type (*arboreal*, *rock climbing*, *terrestrial*, *digging* and *semiaquatic*) and relatedness (i.e., Order). We predict that foot morphology will differ with Locomotion type in mammals. We will discuss our findings and their implications for a bio-inspired climbing shoe design. In particular, we showcase here a method to systematically survey biological solutions to gather a range of ideas prior to generating bio-inspired design concepts.

## 2. Materials and Methods

### 2.1. Photographing of Samples

**Figure 1:**
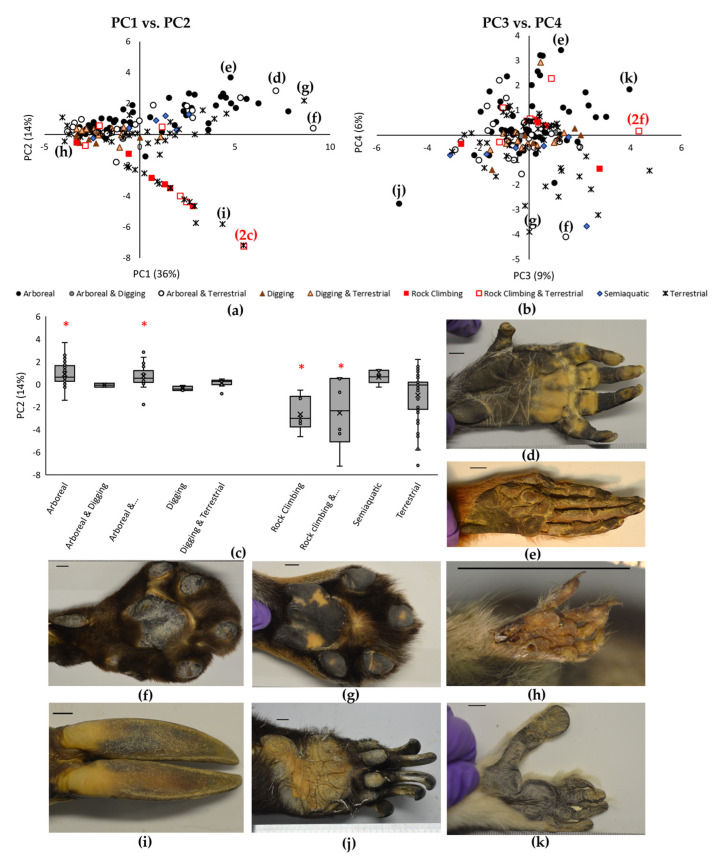
Scattergraph of PCA results, grouped by Locomotion type (**a**,**b**). Locomotion type significantly affects PC1, PC2 and PC3; however when the data is phylogenetically controlled there is not a significant affect. (**c**) Box plot of PC2 grouped by Locomotion type, showing significant differences (*p* < 0.05) using classic ANOVA tests between *Arboreal* and *Arboreal & Terrestrial*, with *Rock Climbing, Rock Climbing & Terrestrial* (indicated with red asterisks). The labels in brackets in (**a**,**b**) correspond to the images in panels (**d**,**k**) here and in Figure 2b,c, showing examples of front and rear feet of the extreme values of the PCA. (**d**) Mandrill, *Mandrillus Sphinx*, (**e**) Bolivian red howler, *Alouatta Sara*, (**f**) Leopard, *Panthera pardus*, (**g**) Tiger, *Panthera tigris*, (**h**) Slender-tailed dunnart, *Sminthopsis Murina,* (**i**) Sitatunga, *Tragelaphus spekii*, (**j**) Sun bear, *Helarctos malayanus*, (**k**) Crowned sifaka, *Propithecus Verrauxi Coronatus*. Scale bar is 10 mm.

The feet of 260 mammalian specimens were photographed. These represent ~5% of all described mammalian species. Specimens were from the skins collections at Liverpool World Museum and National Museums Scotland and specific specimen numbers (when present) can be found in the Supplementary Material (SupplementaryData.pdf). Types of specimens were selected to be proportional to the distribution of mammalian diversity (i.e., more species were sampled from Primates and Rodentia than Artiodactyla). All work was approved by the ethics committee at Manchester Metropolitan University (No: 10682). The objective was to photograph specimens from a range of different genera to ensure diverse examples, since it was observed that mammals from the same genus tended to have similar foot morphology (i.e., see Figure 1f and g for comparison—Leopard, Panthera pardus and Tiger, Panthera tigris). The best specimens available for each genus were selected for photographing, which were identified as the cleanest and best-preserved examples. A digital camera (D3200 with an AF Micro Nikkor 60 mm lens, Nikon, Tokyo, Japan) was positioned above the specimen on a tripod. Photographs were taken with a ruler in focus at the same level of the feet for callibration. A desk lamp was used for photographs when extra light was required.

### 2.2. Image Analysis

All photographs were visually checked to ensure they met the minimum requirement for analysis—the image had to be sufficiently in focus, with minimal visible damage to the specimen, so as not to impact measurements, digit counts or categorisations. When there were multiple photographs of a given foot of a specimen, the best one was selected. Images which fell short of the selection criteria were discarded. The final dataset consisted of 166 species with images of both the front and rear feet of each specimen (332 images in total). This image dataset represented 18 orders, 63 families and 148 genera (~3.5% of all described mammalian species).

Manual analysis of the images was conducted using ImageJ software [37] and a tablet with a pen (PTH-660, Wacom Co., Ltd., Kazo, Saitama, Japan). Calibrated measurements (in mm) were taken on the front and rear foot to give specific lengths and widths, with tracing around features, such as pads, to give areas. These measurements were recorded in a spreadsheet (Excel, Microsoft, Washington, DC, USA) and used to calculate key metrics (Table 1, and Figure S1 in the Supplementary Materials (SupplementaryMethods.pdf)). To gauge uncertainty in these manual measurements, a length (front *Foot length*) and area (front foot area with digits) measurement were repeated three times each on 18 images, corresponding to one species from each Order in the dataset. Differences between the mean of the three repeat measurements and the original one were calculated. Across all 18 images, we found median differences of 1.6 % (range: 0.1–4.5%) for area and 1.70% for length (range: 0.3–15.2%), which suggested that the method was likely to be sufficiently reliable and repeatable.

### 2.3. Data Analysis

Given the age of the specimens, sometimes they were damaged/degraded and digits were hidden. There were also instances where the image focus was compromised due to the different depths of the specimen. In cases where a measurement could not be taken from a certain region of the foot, the mean value of that metric for the dataset was used, so as not to have an affect on the subsequent principal component analysis (PCA). For images where a part of the specimen was out of focus, if pads could be seen elsewhere, they were assumed to also be present on the out of focus section. This was typical with the specimens that had most of the digits in focus but not all, so hidden pads were still included in counts since different digits tended to have the same number of pads.

While hooves are biologically digits, we felt that they offer the only contact point of the foot to the substrate, and were therefore assumed to be more functionally similar to a palmer surface [28]. As such, we treated hooves as anterior pads and allocated zero values to the digit metrics for both the front and rear feet. These metrics included *Mean digit length*, *Mean digit width*, *Digit pad number*, and *Total pad area on digit*, as well as the posterior pad metrics *Total posterior pad area* and *Posterior pad number*. We also left blank the *Digit pad position*, as well as *Posterior pad position*, *Posterior pad texture* and *Posterior pad shape*. We measured the whole hoof area as the *Total anterior pad area* and counted the number of sections to the hoof as part of both the *Anterior pad number* and *Digit number* scores. The *Proportion of digit area* was given the mean value from the dataset so as not to affect the subsequent PCA. See Figure S1 (SupplementoryMethods.pdf) for examples of quantitative metric measurements.

Data for all the quantitative metrics (Table 1) for both front and rear feet, including the *Proportion of digit area*, *Foot length*, *Foot width*, *Mean digit length*, *Mean digit width, Digit number*, *Digit pad number*, *Digit pad area*, *Posterior pad number*, *Total posterior pad area*, *Anterior pad number*, and *Total anterior pad area* were inputted into MATLAB (Version 9.12.0, MathWorks, Massachusetts, MA, USA), normalised, and then a PCA was conducted. The generated PCA ‘scores’ were assigned to each species (i.e., each specimen), so each one was allocated a score for the first four principal components (PC 1–4). Each species was also allocated to a group for Locomotion type and Order. Locomotion type (*Arboreal*, *Arboreal & Terrestrial*, *Arboreal & Digging*, *Digging, Digging & Terrestrial*, *Rock Climbing*, *Rock Climbing & Terrestrial*, *Semiaquatic* and *Terrestrial*) was identified for each specimen by internet searching for key words (Species common name AND climbing OR arboreal OR rock OR digging OR burrowing OR aquatic OR terrestrial) and checking with online resources e.g., the Animal Diversity Web from the University of Michigan [38] and the Invasive Species Compendium [39]. As there is inherent subjectivity associated with assigning Locomotion types, initial allocations were checked and finalised by a second reviewer.

Multivariate ANOVA tests were conducted in r (version 4.2.1, R Core Team and the R Foundation for Statistical Computing, Vienna, Austria) to investigate the association of summary foot morphology metrics from the PCA (PC 1–4) with groupings of Locomotion type and Order. Tukey’s pairwise comparisons were also conducted. ANOVA tests assume that each specimen is independent, and while this is this case, more related species are likely to be more similar. Therefore, an ANOVA was also conducted controlling for phylogeny in r, using the package phytools [40], and a consensus ultrametric tree constructed from 1000 trees from Vertlife.org (downloaded 06/10/22, see Figure S3 in the Supplementary Material (SupplmentaryMethods.pdf)), based on Upham et al. [41]. We also investigated the phylogenetic signal of our PCA data, which is the tendency for related species to resemble each other more than species drawn at random from the same tree. The strength of phylogenetic signal present was calculated across the 1000 trees as Pagel’s lambda (λ), using the phylosig function of phytools. We assumed the measures all followed the expectations of Brownian motion modeling (0 < λ < 1). A likelihood ratio test evaluated whether λ was significantly different from zero. A strong phylogenetic signal, indicating that the trait is evolving by Brownian motion, is indicated by a λ-value close to 1 and a *p*-value < 0.05.

The categorical data for pad texture, shape and position was also investigated. It was grouped by trait (Locomotion type and Order) in percentage frequency plots, and chi-squared tests were conducted in r to investigate associations.

## 3. Results

### 3.1. Principal Component Analysis of Quantitative Metrics

PC1 contributed to 36% of the variance of the data, with PC2 contributing 14%, PC3 9% and PC4 6% (giving a total of 65%) (Table 2). Variable loadings (≥±0.30) indicated, PC1 was mostly associated with front *Foot length* and both front and rear *Foot width*. PC2 was most associated with *Digit number* and *Total anterior pad number*, for both the front and rear feet. PC3 was associated with the *Proportion of digit area*, for both the front and rear feet, and PC4 with *Digit pad number*, for both the front and rear feet, and *Posterior pad number*, for the rear feet (Table 2).

**Figure 2:**
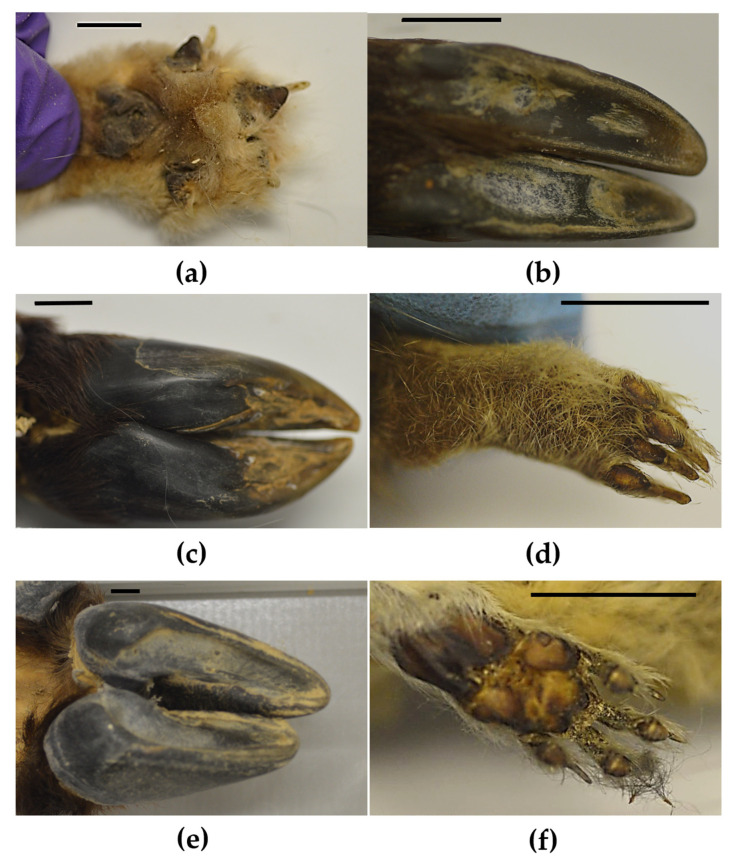
Example pad photographs. (**a**) Blanford’s fox, *Vulpes Cana*, (**b**) Siberian musk deer, *Moschus moschiferus*, (**c**) Yak, *Bos grunniens*, (**d**) Steppe Pika, *Ochtona pusilla*, (**e**) Takin, *Budorcas*, (**f**) Common Gundi, *Ctenodactylus Gundi*. Panels a-b correspond with *Rock Climbing* species, whereas panels (**c**–**f**) correspond with *Rock climbing & Terrestrial* species. Scale bar is 10 mm.

Some PC values significantly varied with Locomotion type (Table 3). In particular, the *Arboreal* and *Arboreal & Terrestrial* species had larger PC2 values than the *Rock Climbing* and *Rock Climbing & Terrestrial* species (Figure 1a,c, Table 3), suggesting that the tree climbers (Figure 1d,e) had more digits and smaller anterior pad areas than the rock climbers (Table 2, Figure 2a,d,f). The *Arboreal* species also had larger PC4 values than the *Terrestrial* species (Figure 1b, Table 3), perhaps indicating that the *Arboreal* species had more pads (digit and posterior pads, Figure 1e,k) than the *Terrestrial* species (Figure 1g) (Table 2). Locomotion type did not have a significant association with PC1 nor PC3 (Figure 1a,b, Table 3). Phylogenetically-controlled ANOVAs did not find significant associations of Locomotion type with PC values (Table 3).

**Figure 3:**
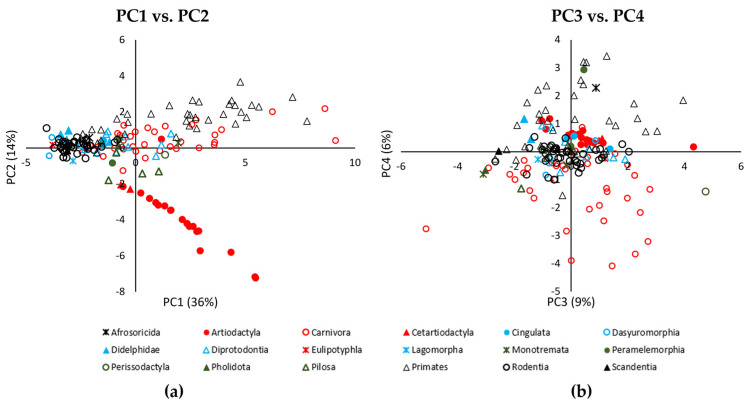
Scattergraph of PCA results, grouped by Order (**a**,**b**). Order had a significant effect on all PC values (1–4).

Indeed, all PC values showed significant effects of Order, with clear groupings of more related species (Figure 3). This is backed up by the finding that all PC values had a significant phylogenetic signal, with all lambda (λ) values >0.75, and all *p*-values < 0.001 (Table 3), indicating that the more related species did, indeed, have more similar foot morphologies. This is further supported by our ANOVA statistical comparisons with Order (Table 3). The Primates and Artiodactyla had significantly higher PC1 values than the Rodentia and Dasyuromorphia, who had relatively low PC1 values (Figure 3a & Table 3). Considering the PC loadings (Table 2), this probably means that the Primates and Artiodactyla had longer and wider feet. The Artiodactyla PC2 values were significantly lower than all other orders apart from the Cetartiodactyla (Figure 3a & Table 3), probably due to the Artiodacyla having hooves; therefore, being allocated a low score for *Digit number* and having a large *Total anterior pad area* (Table 2), as this was measured over the whole hoof surface. The Perissodactyla had a large PC3 value, indicating that it had proportionally larger digit areas than those in many other Orders (Figure 3b, Table 2 and Table 3). The Primates had large PC4 values, which were significantly higher than the Carnivora and Rodentia (Figure 3b & Table 3). Indeed, the Carnivora had particularly low PC4 values, significantly less than the Primates, Artiodactyla, Peramelemorphia, Afrosoricida, Diprodrontia and Didelphimorphia (Figure 3b & Table 3). This suggests that the Carnivora had fewer pads on their digits and on the posterior of their feet (Figure 1f,g) than the Primates (Figure 1d,e) (Table 2).

### 3.2. Pad Positions, Shapes and Textures

**Figure 4:**
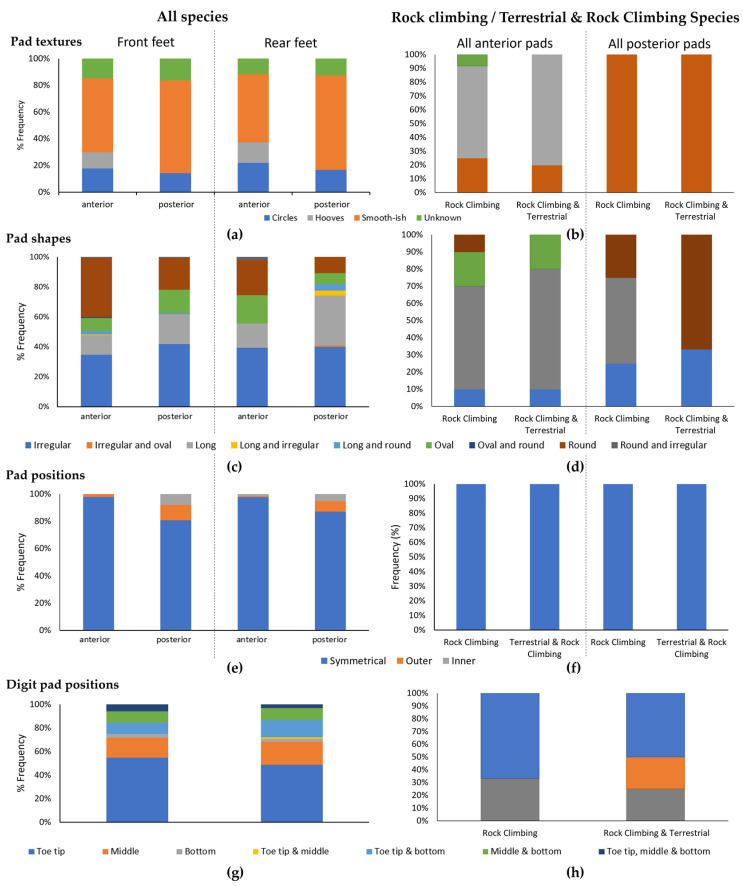
Summary figures of pad positions and textures showing % frequency of occurrence of: (**a**,**b**) Pad textures; (**c**,**d**) Pad shapes; (**e**,**f**) Pad positions; and (**g**,**h**) Digit pad positions. Panels on the left show results from all species, showing the front and rear feet, and both anterior and posterior pads separately. Panels on the right show just Rock Climbing or Rock Climbing & Terrestrial species, and combined data for front and rear feet, with anterior and posterior pads separated.

Pad textures and shapes were relatively variable between species, and are summarised in the left-hand panels in Figure 4. Overall, smooth-ish (55–71%) and circles (14–22%) were the most common pad textures (Figure 4a), and pads were often irregularly shaped (35–42%), round (11–40%) or long (13–33%) (Figure 4c). Pads tended to be positioned fairly symmetrically on both sides of the foot (80–98%) (Figure 4e) with pads on the digit tending to be mainly at the tip (49–55%) (Figure 4g). Despite these overall trends, aspects of pad texture, shape and position varied according to Locomotion type and Order (see Table 4 for detailed statistical analyses).

We now look specifically to species scored as *Rock Climbing* or *Rock Climbing & Terrestrial* in their Locomotion type category (right-hand panels in Figure 4). On the whole, these species had hooves (Figure 2b,c,e) or smooth-ish pads (Figure 2a,d,f). Circular textured pads were absent in these species. If the species had pads, they could be long, oval, irregular and round anteriorly, and round, irregular and long posteriorly (Figure 4d). When present, pads were always positioned symmetrically on both sides of the foot (Figure 2a,d,f and Figure 4f). Digit pads, when present, could be at the toe tip, middle and bottom of the digits of *Rock Climbing & Terrestrial* species, and were less variable in *Rock Climbing* species, only appearing at the toe tip and bottom of the digit (Figure 4h).

## 4. Discussion

This study showcases the first step of a bio-inspired design process, to systematically survey biological solutions, to gather a range of ideas before generating design concepts. It is the first study to describe foot morphology over a diverse group of mammals. To do so, we developed a new approach to measure and categorise foot morphology in mammals, which was sensitive enough to reveal differences between certain groups of species. As predicted, Locomotion type affected aspects of mammalian foot morphology. Species relatedness (Order) also strongly affected foot morphology (i.e., all λ values > 0.75 and *p* values < 0.001). When we summarised our quantitative metrics of foot morphology using PCA, we found that PC2 (describing ~15% of variation in the data) differed between rock climbing species (including those coded as *Rock Climbing* and *Rock Climbing & Terrestrial*), compared to tree climbing species in our sample (including *Arboreal*, and *Arboreal & Terrestrial*). Specifically, this suggests that the rock climbing species had fewer digits and larger anterior pad areas. Indeed, the rock climbing species often had hooved feet and if they had pads, they tend to be smooth-ish (Figure 2).

The presence of many, small foot pads (especially in the anterior region), indicated by large PC2 and PC4 values (Figure 1), were present in arboreal mammals (including *Arboreal*, and *Arboreal & Terrestrial*) within our sample. The presence of many, small foot pads has been documented before in small primates [25] and rodents [31]. The presence of long, deformable digits is also associated with arboreality, particularly for grasping during tree climbing in primates and rodents [25,31]. Rock climbing species in our sample had fewer digits and larger anterior pads than tree climbing species (Table 2 and Table 3), which highlights the importance of focusing on rock climbing animals during a survey of this nature, rather than simply looking generally at animals that climb. The fewer digits in rock climbing species might reflect the typically large, inclined surfaces of rocks, which are dissimilar to the smaller branches that can be grasped during tree climbing. Having larger, fewer pads (or fused pads), has only been observed before in larger and heavier arboreal animals (i.e., in large primates and humans [25,42]). Large pads are thought to provide a uniform traction surface for tree climbing [30,42], and are likely to also provide a similar function in these rock climbing animals.

Labonte and Federle [30] state that larger animals can compensate for their weight during climbing by having larger pads, as well as increasing attachment efficiency (i.e., friction per unit contact area). We notice that the rock climbing animals in our sample, overall, had smooth-ish pads or hooves (Figure 4). However, even though the foot surfaces may appear largely smooth, there were differences in surface texture (see Figure S2 (SupplementaryMethods.pdf)). These differences were hard to categorise further, as texture varied upon a continuum. Even small changes in surface texture can increase friction. For example, even the small ridges on primate and human volar skin (i.e., the fingertip ridges) may increase friction during climbing (see Figure S2b (SupplementaryMethods.pdf)) [25]. And even hooves (e.g., Figure 2b,c,e), which appear to be smooth, have a textured surface, which is thought to increase friction with smooth surfaces and also absorb shocks [28]. Therefore, surface area, texture, compliance, and friction are all likely to aid climbing.

Although, on the whole, rock climbing species had few digits, and large smooth-ish pads or hoof-like feet, there was still some variation, especially in pad shape and position (Figure 2). Much of this variation is likely to be dependent on the relatedness of species, with those that are more related having more similar feet, irrespective of their Locomotion type. This would explain why our phylogenetically-corrected ANOVA had no significant effect of Locomotion type on PC values 1–4, whereas our classic ANOVA revealed significant effects in PC1 and PC2 (Table 3). Indeed, Order had a significant effect on PC1–4, with the orders Primates, Artiodactyla and Rodentia often differing from the others (Table 3). Within each of the orders, Locomotion types varied. For example, rock climbing (including *Rock Climbing & Terrestrial*) species were present in the orders Carnivora (e.g., foxes), Artiodactyla (e.g., bovids and goats), Rodentia (e.g., gundis or comb rats), Afrosoricida (e.g., tenrecs) and Lagomorpha (e.g., pika) (see examples in Figure 2). Therefore, perhaps it would be beneficial to consider innovations from each Order, when considering rock climbing feet adaptations for bio-inspired footwear design, since between-Order effects were large in our sample.

While we took care to proportionally sample from each Order, we have only captured a small selection (~3.5%) of mammalian species. Therefore, more adaptations in foot morphology may exist than what we have characterised here. However, we have represented many different mammalian Orders, and our results do suggest that between-Order effects are large, compared to within-Order effects (i.e., from our Phylogenetic statistics). Therefore, we may not expect to see many further extremely distinct examples of foot morphology if we were to sample from more species. Furthermore, this study represents the largest comparison of foot morphologies in mammals, and the largest biological comparison (to our knowledge) for bio-inspired design purposes.

### Implications for Bio-Inspired Climbing Shoe Design

**Figure 5:**
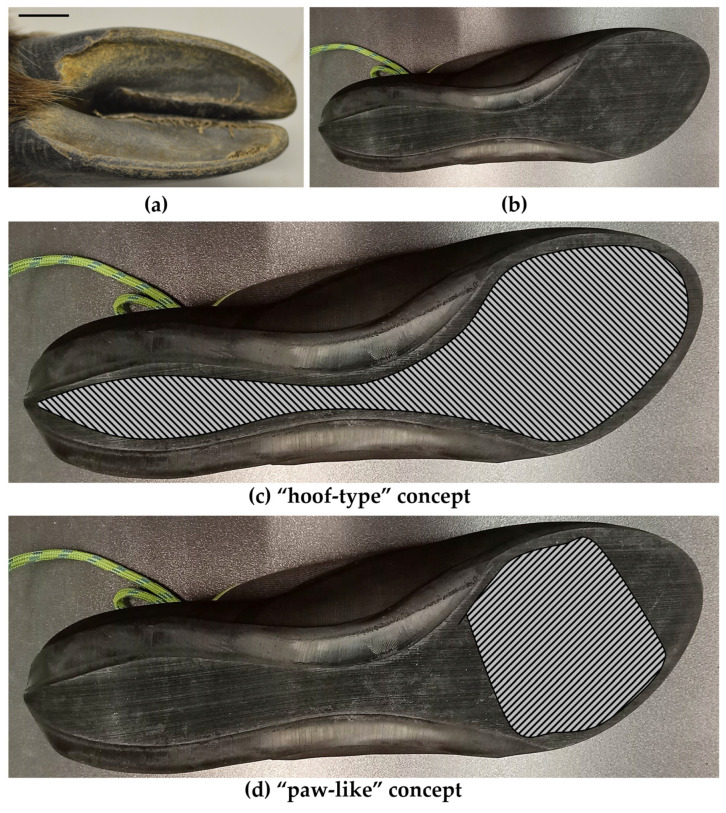
Example foot image of *Rock Climbing & Terrestrial* species Mainland Serow *(Capricornis sumatraensis)* (**a**) compared to an example climbing shoe (**b**). Example designs might include inserting a compliant or textured surface into the full sole (hatched area on photograph), like a hoof (**c**), or in large anterior pads, similar to a paw-like foot of a rock climbing mammal (**d**). Texture, compliance and position of these structures will need further development and testing before full candidate designs can be proposed. Scale bar in panel a is 10 mm.

There is some variation within the foot morphology of climbing mammals, however, most species we sampled had large, smooth-ish foot pads or hooves (Figure 4b). Indeed, the hooved feet of many rock climbing mammals look somewhat superficially like current climbing shoes (Figure 5a,b). Therefore, perhaps climbing shoe designs have convergently developed, or “evolved”, to be relatively similar to the feet of rock climbing mammals. However, while there are some similarities, we do believe that there is room for further innovations, taking inspiration from rock climbing mammals (Table 5). The presence of pads on some climbing species, suggests that having a compliant structure might be useful during rock climbing, especially a large anterior pad (i.e., Figure 5d).

Compliant pads have also been documented in hooves (e.g., in goats). Although the edge of the hoof is made of stiff keratin, the central area is compliant for stabilization and to absorb shocks [28]. This palmar region is also slightly textured to increase friction with a surface [28]. Therefore, a compliant and textured sole surface might be a useful addition to climbing shoes (i.e., Figure 5c or d). However, texture, and shape needs to be better captured and described in our samples before fully developing these bio-inspired designs, perhaps by using three-dimensional imaging techniques, such as photogrammetry and structured light scanning. Indeed, future work could focus on characterizing these features on the feet of climbing animals in more detail, to aid the development of candidate shoe design concepts. Overall, incorporating surface textures and compliant pads into a climbing shoe may well improve grip and traction with the climbing surface, as well as improving stabilization and shock absorbance. The texture, compliance and position of these structures will need further characterization and testing before full candidate design concepts can be proposed. These concepts could then be tested experimentally, such as with tests of friction (e.g., [43]) and compliance, to better determine those which could be applied to improve climbing shoe designs. These tests should, ideally, be representative of the forces being applied to the shoe sole in the same way that they are applied to the mammalian foot during rock climbing.

## 5. Conclusions

We suggest that a systematic survey of biological examples, such as we have conducted here, will help to solve design problems using true inspiration from nature. While many bio-inspired studies appear to focus on only one biological solution, we show that there may be an array of biological solutions to a common problem, which in this case is rock climbing. This study showcases the first step in a bio-inspired design process, to systematically explore and describe biological examples. This step may include comparing specific biological solutions to those of other traits. For example, here we find the adaptations of rock climbing mammalian feet by comparing them with species that undertake other locomotion strategies. While comparisons are important to highlight adaptations, they also need to be specific and appropriate. For example, in our study, tree climbing and rock climbing animals had distinctly different foot morphologies, so it would be inappropriate to base a rock climbing shoe on an arboreal mammalian foot. The next step in the design process, would be to: (i) further quantify and test the functional properties of these adaptations (i.e., pad texture and compliance), and then (ii) develop and test candidate design concepts. We feel that such a systematic survey of biological examples will lead to a more stringent approach to bio-inspired design and generate more possible solutions to engineering problems.

## Figures and Tables

**Table 1:** Quantitative and categorical metrics extracted from feet photographs. All metrics were applied to front and rear feet. Figures S1 and S2 in Section S1 of the Supplementary Materials (SupplementaryMethods.pdf).

Measurements	Unit	Description	Figure
*Quantitative metrics*			
Proportion of digit area	-	Difference between the area of the foot with and without the digits, divided by the area of the foot with the digits.^1^	Figure S1a,b
Foot length	mm	Length at longest point of the foot.	Figure S1d
Foot width	mm	Width at widest point of the foot.	Figure S1e
Mean digit length	mm	Mean of the tip to base length of all digits.^1^	Figure S1g
Mean digit width	mm	Mean of the width of the widest point of all digits.	Figure S1h
Digit number	No.	Count of no. digits per foot.	Figure S1f
Digit pad number	No.	Count of total no. pads on digits.	Figure S1f
Total pad area on digit	${\mathrm{mm}}^{2}$	Total area of digit pads.	Figure S1f
Posterior pad number	No.	Count of no. posterior pads.^2^	Figure S1c
Total posterior pad area	${\mathrm{mm}}^{2}$	Total area of posterior pads.	Figure S1c
Anterior pad number	No.	Count of no. anterior pads.^2^	Figure S1i
Total anterior pad area	${\mathrm{mm}}^{2}$	Total area of anterior pads.	Figure S1i
*Categorical metrics*			
Digit pad position		Scored as: toe tip, middle, bottom.^3^	
Anterior pad position		Scored as: inner, outer or symmetrical.^2^	
Posterior pad position		As above.	
Posterior pad shape		Scored as: irregular, long, oval or round.^3^	
Anterior pad shape		As above.	
Posterior pad texture		Scored as: circles, smooth-ish ^4^, or unknown.^5^	Figure S2
Anterior pad texture		Scored as: circles, hooves ^6^, smooth-ish ^4^, or unknown.^5^	Figure S2

^1^ Excluding the claws. ^2^ Anterior and posterior pads were identified as such by eye. Some pads went over both sections of the foot (posterior and anterior), and these were only allocated into one category (posterior or anterior) based on the amount of the pad in each foot section. ^3^ Combinations of these also occurred and were recorded as such. ^4^ Smooth included shallow horizontal grooves (Figure S2b) irregular shallow grooves (Figure S2c) and very smooth pads (Figure S2d). ^5^ The texture was scored as unknown if it was unclear. ^6^ Hooves were scored in the anterior section of the pad only, and not considered in the posterior pad.

**Table 2:** Variable loadings (≥±0.30) for the first four principal components, in descending order.

PC1 (36%)	PC2 (14%)	PC3 (9%)	PC4 (6%)
Front *Foot length* (0.32)	Rear foot *Digit number* (0.46)	Front foot *Proportion of digit area* (0.55)	Rear foot *Digit pad number* (0.52)
Front *Foot width* (0.32)	Front foot *Digit number* (0.44)	Rear foot *Proportion of digit area*	Front foot *Digit pad number* (0.46)
Rear *Foot width* (0.32)	Rear foot *Total anterior pad area* (−0.39)	(0.54)	Rear foot *Posterior pad number* (0.30)
	Front foot *Total anterior pad area* (−0.36)		

**Table 3:** Statistical comparisons of the first four Principal Components (PC), grouped by Locomotion type and Order, and analysed using classic ANOVAs or ANOVAs controlling for phylogenetic relatedness (Phylo ANOVA). Post-hoc tests were conducted using Tukey’s test. Phylogenetic relatedness was investigated using Pagel’s λ. All significant values were *p* < 0.05.

Comparison	Test	F	Df1, Df2	*p*	Post-Hoc
**PC1 (λ = 0.93, *p* < 0.001)**					
Order	ANOVA	9.32	17, 149	<0.001	Primates, Artiodactyla > Rodentia, Didelphimorphia, Dasyuromorphia Rodentia, Dasyuromorphia < Primates, Artiodactyla, Carnivora
Locomotion	ANOVA	1.53	10, 156	0.133	-
Locomotion	Phylo ANOVA	1.36	-	0.800	-
**PC2 (λ = 0.86, *p* < 0.001)**					
Order	ANOVA	32.90	17, 149	<0.001	Artiodactyla < all groups apart from Cetartiodactyla Cetartiodactyla < Primates, Carnivora, Rodentia, Didelphimorphia Primates > Cetartiodactyla, Rodentia, Artiodactyla, Pilosa Rodentia > Cetartiodactyla, Artiodactyla, Pilosa
Locomotion	ANOVA	8.94	10, 156	<0.001	Arboreal & Terrestrial, Arboreal > Rock Climbing, Rock Climbing & Terrestrial
Locomotion	Phylo ANOVA	8.49	-	0.200	-
**PC3 (λ = 0.84, *p* < 0.001)**					
Order	ANOVA	2.07	17, 149	0.011	Perissodactyla > Primates, Carnivora, Rodentia, Didelphimorphia, Monotremata, Pilosa, Pholidota, Scandentia
Locomotion	ANOVA	1.11	10,156	0.360	-
Locomotion	Phylo ANOVA	0.99	-	1.000	-
**PC4 (λ = 0.77, *p* < 0.001)**					
Order	ANOVA	9.94	17, 149	<0.001	Carnivora < Primates, Artiodactyla, Peramelemorphia, Afrosoricida, Diprodrontia, Didelphimorphia Primates > Carnivora, Rodentia
Locomotion	ANOVA	2.53	10, 156	0.008	Arboreal > Terrestrial
Locomotion	Phylo ANOVA	2.18	-	0.900	-

**Table 4:** Statistical associations of foot pad texture, shape, position and digit pad position with Locomotion type and Order. Explored using chi-squared tests of association.

	Front Feet		Rear Feet	
Pads	Anterior	Posterior	Anterior	Posterior
**Locomotion**				
Texture	Χ^2^ = 112.96, df = 32, *p* < 0.001	Χ^2^ = 66.192, df = 24, *p* < 0.001	Χ^2^ = 117.84, df = 32, *p* < 0.001	Χ^2^ = 51.693, df = 24, *p* = 0.001
Shape	Χ^2^ = 83.618, df = 64, *p* = 0.050	Χ^2^ = 74.846, df = 48, *p* = 0.008	Χ^2^ = 68.82, df = 48, *p* = 0.026	Χ^2^ = 83.424, df = 56, *p* = 0.026
Position	Χ^2^ = 27.841, df = 16, *p* = 0.033	Χ^2^ = 79.033, df = 32, *p* < 0.001	Χ^2^ = 42.224, df = 24, *p* = 0.012	Χ^2^ = 47.076, df = 32, *p* = 0.042
Digit position	Χ^2^ = 108.85, df = 48, *p* < 0.001		Χ^2^ = 105.44, df = 56, *p* < 0.001	
**Order**				
Texture	Χ^2^ = 272.4, df = 68, *p* < 0.001	Χ^2^ = 218.68, df = 51, *p* < 0.001	Χ^2^ = 312.47, df = 68, *p* < 0.001	Χ^2^ = 145.79, df = 51, *p* < 0.001
Shape	Χ^2^ = 247.1, df = 136, *p* < 0.001	Χ^2^ = 213.06, df = 102, *p* < 0.001	Χ^2^ = 207.88, df = 102, *p* < 0.001	Χ^2^ = 197.85, df = 119, *p* < 0.001
Position	Χ^2^ = 79.123, df = 34, *p* < 0.001	Χ^2^ = 188.09, df = 68, *p* < 0.001	Χ^2^ = 87.954, df = 51, *p* = 0.001	Χ^2^ = 107.22, df = 68, *p* = 0.002
Digit position	Χ^2^ = 367.25, df = 102, *p* < 0.001		Χ^2^ = 525.27, df = 119, *p* < 0.001	

**Table 5:** Recommendations for bio-inspired climbing shoe designs based on our findings.

Biological Finding	Bio-Inspired Design Recommendation
Hooves or large pads common	Characterise compliance of the pads and hooves and create candidate concepts for testing, varying in compliance
Smooth-ish pads and hooves, but some texture present	Characterise texture of the pads and hooves and create candidate texture concepts for testing
Variation in pad location and shape	Test candidate pad structures with texture and compliance over a range of likely positions on the shoe
Order effects foot morphology	Choose representative candidate concepts based on a range of rock climbing Orders (Carnivora, Artiodactyla, Rodentia, Afrosoricida and Lagomorpha)

## Data Availability

Summary metrics of the data presented in this study are openly available in the supplementary material of this manuscript. Photographic images captured in this study are available on request from the corresponding author.

## Supplementary Materials

The following supporting information can be downloaded at: https://www.mdpi.com/article/10.3390/biomimetics8010008/s1, Figure S1: Summary of measurements from photographs for a paw-type foot and a hoof-type foot; Figure S2: Summary of pad textures; Figure S3: Phylogenetic consensus tree used for Phylogenetic ANOVA statistics. Table S1: Supplementary Data.
