# Supplementary material for: Investigating Foot Morphology in Rock Climbing Mammals: Inspiration for Biomimetic Climbing Shoes
Source: Biomimetics (Basel). 2022 Dec 24;8(1):8. doi: 10.3390/biomimetics8010008 (PMC9844278; doi:10.3390/biomimetics8010008)
Supplement: Supplementary file 1 [file biomimetics-08-00008-s001.zip › SupplementaryData.pdf]

Investigating foot morphology in rock climbing mammals: inspiration for biomimetic climbing shoes

	Scientific name	Common Name	Order
1	Varecia_variegata	Black-and-white ruffed lemur	Primates
2	Eulemur_mongoz	Mongoose lemur	Primates
3	Cheirogaleus_medius	Fat-tailed dwarf lemur	Primates
4	Galago_senegalensis	Southern needle-clawed bushbaby	Primates
5	Propithecus_verreauxi	Crowned sifaka	Primates
6	Avahi_laniger	Eastern woolly lemur	Primates
7	Callithrix_jacchus	Common marmoset	Primates
8	Mico_argentatus	Silvery marmoset	Primates
9	Callimico_goeldii	Goeldi's monkey	Primates
10	Leontopithecus_rosalia	Golden lion tamarin	Primates
11	Saguinus_oedipus	Cotton-top tamarin	Primates
12	Sapajus_apella	Tufted capuchin	Primates
13	Ateles_fusciceps	Black-headed spider monkey	Primates
14	Lagothrix_poeppigii	Silvery woolly monkey	Primates
15	Pithecia_pithecia	Saki Monkey	Primates
16	Alouatta_sara	Bolivian red howler	Primates
17	Aotus_lemurinus	Gray-bellied night monkey	Primates
18	Cercopithecus_lhoesti	L'Hoest's monkey	Primates
19	Cercopithecus_diana	Diana monkey	Primates
20	Erythrocebus_patas	Patas monkey	Primates
21	Allenopithecus_nigroviridis	Allen's swamp monkey	Primates
22	Macaca_nigra	Celebes crested macaque	Primates
23	Theropithecus_gelada	Gelada	Primates
24	Papio_hamadryas	Hamadryas baboon	Primates
25	Mandrillus_sphinx	Mandrill	Primates
26	Colobus_guereza	Mantled guereza	Primates
27	Procolobus_preussi	Preuss's red colobus	Primates
28	Presbytis_melalophos	Black-crested Sumatran langur	Primates
29	Trachypithecus_francoisi	François' langur	Primates
30	Semnopithecus_entellus	Northern plains gray langur	Primates
31	Nomascus_leucogenys	Northern white-cheeked gibbon	Primates
32	Hylobates_pileatus	Pileated gibbon	Primates
33	Symphalangus_syndactylus	Siamang	Primates
34	Pan_paniscus	Bonobo	Primates
35	Tapirus_terrestris	South American tapir	Perissodactyla
36	Pudu_puda	Pudú	Cetartiodactyla
37	Tragulus_javanicus	Java mouse-deer	Cetartiodactyla
38	Vicugna_vicugna	Vicuña	Artiodactyla
39	Mustela_nivalis	Weasel	Carnivora
40	Vormela_peregusna	Marbled polecat	Carnivora
41	Neovison_vison	American mink	Carnivora

42	Nasua_nasua	South American coati	Carnivora
43	Mydaus_javanensis	Sunda stink badger	Carnivora
44	Ailurus_fulgens	Red panda	Carnivora
45	Meles_meles	Badger	Carnivora
46	Martes_pennanti	Fisher	Carnivora
47	Ictonyx_striatus	Polecat	Carnivora
48	Martes_martes	European pine marten	Carnivora
49	Lutra_lutra	Eurasian otter	Carnivora
50	Aonyx_cinerea	Asian small-clawed otter	Carnivora
51	Lontra_canadensis	North American river otter	Carnivora
52	Enhydra_lutris	Sea otter	Carnivora
53	Arctonyx_collaris	Hog badger	Carnivora
54	Helarctos_malayanus	Sun bear	Carnivora
55	Vulpes_vulpes	Red fox	Carnivora
56	Ursus_arctos	Brown bear	Carnivora
57	Canis_lupus	Dog	Carnivora
58	Canis_lupus	Wolf	Carnivora
59	Chrysocyon_brachyurus	Maned wolf	Carnivora
60	Cuon_alpinus	Dhole	Carnivora
61	Lycaon_pictus	African wild dog	Carnivora
62	Speothos_venaticus	Bush dog	Carnivora
63	Nyctereutes_procyonoides	Raccoon dog	Carnivora
64	Panthera_tigris	Tiger	Carnivora
65	Panthera_pardus	Leopard	Carnivora
66	Paradoxurus_hermaphroditus	Asian palm civet	Carnivora
67	Arctictis_binturong	Bearcat	Carnivora
68	Arctogalidia_trivirgata	Small-Toothed Palm civet	Carnivora
69	Proteles_cristata	Aardwolf	Carnivora
70	Herpestes_sanguineus	Common Dwarf Moongoose	Carnivora
71	Nandinia_binotata	African palm civet	Carnivora
72	Atilax_paludinosus	Marsh mongoose	Carnivora
73	Cryptoprocta_ferox	Fossa	Carnivora
74	Vulpes_cana	Blanford's fox	Carnivora
75	Moschus_moschiferus	Siberian musk deer	Artiodactyla
76	Tayassu_pecari	Collared peccary	Artiodactyla
77	Equus_grevyi	Grévy's zebra	Artiodactyla
78	Axis_kuhlii	Bawean deer	Artiodactyla
79	Okapia_johnstoni	Okapi	Artiodactyla
80	Eudorcas_albonotata	Mongalla gazelle	Artiodactyla
81	Bos_grunniens	Yak	Artiodactyla
82	Addax_nasomaculatus	Addax	Artiodactyla
83	Ovibos_moschatus	Muskox	Artiodactyla
84	Hemitragus_jemlahicus	Arabian tahr	Artiodactyla
85	Naemorhedus_caudatus	Long-tailed goral	Artiodactyla
86	Capra_aegagrus	Goat	Artiodactyla
87	Capricornis_sumatraensis	Mainland serow	Artiodactyla
88	Tragelaphus_spekii	Sitatunga	Artiodactyla

89	Ammotragus_lervia	Barbary sheep	Artiodactyla
90	Budorcas_taxicolor	Takin	Artiodactyla
91	Oryx_leucoryx	Arabian oryx	Artiodactyla
92	Kobus_megaceros	Nile lechwe	Artiodactyla
93	Petaurista_magnificus	Hodgson's giant flying squirrel	Rodentia
94	Petaurista_petaurista	Red Giant Flying Squirrel	Rodentia
95	Paraxerus_boehmi	Green bush squirrel	Rodentia
96	Paraxerus_poensis	boehm's bush squirrel	Rodentia
97	Marmota_himalayana	Himalayan Marmot	Rodentia
98	Iomys_horsfieldii	Javanese Flying Squirrel	Rodentia
99	Heliosciurus_rufobrachium	red-legged sun squirrel	Rodentia
100	Heliosciurus_gambianus	Gambian Sun Squirrel	Rodentia
101	Funisciurus_pyrropus	Fire footed rope squirrel	Rodentia
102	Funisciurus_carruthersi	Curruther's Mountain Squirrel	Rodentia
103	Funambulus_pennantii	Northern Palm Squirrel	Rodentia
104	Callosciurus_prevostii	Prevost's Squirrel	Rodentia
105	Callosciurus_notatus	Plantain Squirrel	Rodentia
106	Ammospermophilus_leucurus	White-Tailed Antelope Squirrel	Rodentia
107	Ratufa_indica	Indian Giant Squirrel	Rodentia
108	Ratufa_bicolor	Black Giant Squirrel	Rodentia
109	Sciurus_carolinensis	Eastern Grey Squirrel	Rodentia
110	Sciurus_colliaei	Collie's squirrel	Rodentia
111	Sundasciurus_tenuis	Slender squirrel	Rodentia
112	Urocitellus_undulatus	Barrow ground squirrel	Rodentia
113	Xerus_inauris	Cape ground squirrel	Rodentia
114	Sciurus_spadiceus	Southern Amazon red squirrel	Rodentia
115	Tamiasciurus_hudsonicus	American Red squirrel	Rodentia
116	Thomomys_umbrinus	Southern pocket gopher	Rodentia
117	Geomys_bursarius	Plains pocket gopher	Rodentia
118	Dendrolagus_bennettianus	Tree-kangaroo	Diprotodontia
119	Macropus_agilis	Agile wallaby	Diprotodontia
120	Trichosurus_vulpecula	Common brushtail possum	Diprotodontia
121	Pseudocheirus_peregrinus	Common ringtail possum	Diprotodontia
122	Phalanger_orientalis	Phalanger	Diprotodontia
123	Macrotis_lagotis	Greater bilby	Peramelemorphia
124	Marmosa_robinsoni	Robinson's mouse opossum	Didelphimorphia
125	Sminthopsis_murina	Slender-tailed dunnart	Dasyuromorphia
126	Philander_opossum	Gray four-eyed opossum	Didelphimorphia
127	Dasyurus_viverrinus	Eastern quoll	Dasyuromorphia
128	Didelphis_virginiana	Virginia opossum	Didelphimorphia
129	Tachyglossus_aculeatus	Short-beaked echidna	Monotremata
130	Ornithorhynchus_anatinus	Platypus	Monotremata
131	Dasypus_novemcinctus	Armadillo	Cingulata
132	Cyclopes_didactylus	Silky anteater	Pilosa
133	Tamandua_tetradactyla	Southern tamandua	Pilosa
134	Trichosurus_vulpecula	Common brushtail possum	Peramelemorphia
135	Thylogale_thetis	Red-necked pademelon	Diprotodontia

136	Wallabia_bicolor	Swamp wallaby	Diprotodontia
137	Phascolarctos_cinereus	Koala	Diprotodontia
138	Myrmecophaga_tridactyla	Giant anteater	Pilosa
139	Smutsia_temminckii	Ground pangolin	Pholidota
140	Chaetophractus_vellerosus	Screaming Hairy Armadillo	Cingulata
141	Choloepus_didactylus	Linnaeus's two-toed sloth	Pilosa
142	Mus_musculus	House Mouse	Rodentia
143	Ctenodactylus_gundi	Common Gundi	Rodentia
144	Rattus_rattus	Black Rat	Rodentia
145	Sorex_araneus	Common Shrew	Eulipotyphla
146	Tupaia_tana	Large Treeshrew	Scandentia
147	Tenrec_ecaudatus	Tailless Tenrec	Afrosoricida
148	Peromyscus_maniculatus	Eastern Deer mouse	Rodentia
149	Petaurus_breviceps	Sugar Glider	Diprotodontia
150	Antechinus_flavipes	Yellow-footed Antechinus	Dasyuromorphia
151	Ochotona_pusilla	Steppe Pika	Lagomorpha
152	Pan_troglodytes	Chimpanzee	Primates
153	Otospermophilus_beecheyi	California ground squirrel	Rodentia
154	Marmota_monax	Groundhog	Rodentia
155	Pteromys_volans	Siberian flying squirrel	Rodentia
156	Macropus_irma	Western brush wallaby	Diprotodontia
157	Orthogeomys_hispidus	Hispid pocket gopher	Rodentia
158	Spermophilus_pygmaeus	Little ground squirrel	Rodentia
159	Tamias_striatus	Eastern chipmunk	Rodentia
160	Microsciurus_flaviventer	Amazon dwarf squirrel	Rodentia
161	Tamias_townsendii	Townsend's chipmunk	Rodentia
162	Thomomys_mazama	Mazama pocket gopher	Rodentia
163	Ictidomys_tridecemlineatus	Thirteen-lined ground squirrel	Rodentia
164	Exilisciurus_whiteheadi	Tufted pygmy squirrel	Rodentia
165	Dipodomys_merriami	Meniam Kangeroo Rat	Rodentia
166	Loris_tardigradus	Gray slender loris	Primates

**Family**	**Specimen Number**	**Locomotion**	**PC1**	**PC2**	**PC3**	**PC4**
Lemuridae	158	Arboreal	2.59	2.65	0.44	2.41
Lemuridae	11	Arboreal	-0.50	0.77	-0.11	0.24
Cheirogaleidae	RL02	Arboreal	-2.50	0.55	1.23	0.03
Galagidae	527	Arboreal	-2.42	0.78	0.65	0.09
Indriidae	92	Arboreal	2.88	2.57	3.95	1.84
Indriidae	13 / 67	Arboreal	-0.35	1.43	3.04	0.74
Callitrichidae	427.014	Arboreal	-2.00	1.03	-0.06	0.03
Callitrichidae	50.003	Arboreal	-2.18	0.04	-0.79	0.17
Callitrichidae	61.004	Arboreal	-1.57	1.01	-1.07	0.22
Callitrichidae	277.008	Arboreal	-1.57	0.42	-0.83	1.10
Callitrichidae	126	Arboreal	-1.78	-0.26	-1.91	0.94
Cebidae	PH77.13	Arboreal	-0.38	1.89	0.54	3.19
Atelidae	84	Arboreal	4.80	2.00	-1.13	1.35
Atelidae	072 608	Arboreal	4.00	1.87	0.13	1.16

Pitheciidae	Ph14.14	Arboreal	1.54	2.37	0.44	3.22
Atelidae	901	Arboreal	4.77	3.68	1.26	3.42
Aotidae	187 22	Arboreal	-0.86	1.62	-1.13	1.13
Cercopithecidae	229 001	Arboreal	2.37	1.90	-1.55	1.76
Cercopithecidae	47	Arboreal	4.38	2.64	0.35	2.56
Cercopithecidae	PH182.12	Terrestrial	3.32	1.57	-1.94	-0.29
Cercopithecidae	70	Arboreal	2.30	1.51	-0.49	0.09
Cercopithecidae	119.004	Arboreal	4.53	2.37	0.10	2.01
Cercopithecidae	122.001	Arboreal & Terrestrial	2.78	1.08	-2.68	-0.25
Cercopithecidae	91.002	Terrestrial	4.55	1.34	-2.27	0.08
Cercopithecidae	222.001	Arboreal & Terrestrial	7.15	2.83	-1.62	0.19
Cercopithecidae	179.003	Arboreal	5.05	1.71	-0.87	2.36
Cercopithecidae	89.6	Arboreal	2.73	1.70	2.63	0.73
Cercopithecidae	0.029	Arboreal	1.84	0.59	-0.64	0.77
Cercopithecidae	183	Arboreal & Terrestrial	5.29	2.38	-1.17	2.21
Cercopithecidae	162	Arboreal & Terrestrial	2.48	1.84	-0.90	1.49
Hylobatidae	086.2 / PH103.06	Arboreal	5.61	2.29	1.57	0.94
Hylobatidae	085.1 / PH105.06	Arboreal	4.27	2.44	1.34	1.11
Hylobatidae	085.3 / PH106.06	Arboreal	7.80	1.49	2.34	1.20
Homininae	166	Arboreal & Terrestrial	0.71	1.90	-0.30	-1.56
Tapiridae	112.003	Terrestrial	1.34	-0.38	4.74	-1.42
Cervidae	401	Terrestrial	-0.24	-2.29	0.98	0.40
Tragulidae	PH50.98	Terrestrial	-0.68	-2.05	1.10	0.47
Camelidae	72.2	Terrestrial	2.62	-4.37	-0.02	0.56
Mustelidae	248.011	Arboreal & Terrestrial	-1.48	0.13	1.07	-0.25
Mustelidae	187.008	Terrestrial	-2.06	0.13	2.25	-0.85
Mustelidae	245	Semiaquatic	-2.44	-0.25	1.56	-0.07
Procyonidae	PH63.06	Arboreal & Terrestrial	-0.02	0.89	-2.91	-0.58
Mephitidae		Digging & Terrestrial	-0.97	-0.02	-1.53	-0.59
Ailuridae	39	Arboreal	3.62	0.72	1.00	-1.92
Mustelidae	23	Digging & Terrestrial	1.10	-0.16	-0.29	-0.50
Mustelidae		Arboreal & Terrestrial	-0.23	0.64	-0.01	-0.12
Mustelidae	86.13	Arboreal & Terrestrial	-1.71	0.05	1.99	-0.46
Mustelidae	72	Arboreal	-0.72	0.70	0.15	0.02
Mustelidae	74	Semiaquatic	1.35	1.21	-0.52	-0.51
Mustelidae	174	Semiaquatic	-0.56	0.41	-1.67	-0.76
Mustelidae	GH 3.10 09	Semiaquatic	0.86	0.90	0.58	-0.43
Mustelidae	169.001	Semiaquatic	2.57	1.30	2.26	-3.66
Mustelidae	079. 2/229	Digging	0.02	-0.20	-1.45	-1.39
Ursidae	71	Arboreal	6.24	2.01	-5.12	-2.76
Canidae	30	Terrestrial	0.44	-0.57	1.28	-1.43
Ursidae	EBB / 01RP	Terrestrial	2.89	0.00	-1.30	-1.66
Canidae		Terrestrial	1.59	0.07	1.16	-2.47
Canidae	46.6	Terrestrial	3.92	1.01	2.72	-3.21
Canidae	154	Terrestrial	2.06	0.34	2.44	-2.18
Canidae	PH20.06	Terrestrial	1.86	-0.05	0.65	-2.06
Canidae	127.2 / PH23.00	Terrestrial	3.59	0.18	-0.16	-2.84

Canidae	R285.99	Terrestrial	0.40	0.19	0.39	0.87
Canidae	GH73.14	Terrestrial	-0.56	-0.07	1.99	-1.66
Felidae		Terrestrial	8.64	2.19	0.01	-3.89
Felidae		Arboreal & Terrestrial	9.11	0.41	1.45	-4.09
Viverridae		Arboreal & Terrestrial	-0.72	0.57	-0.66	-0.41
Viverridae		Arboreal	3.60	0.30	-2.16	-0.81
Viverridae		Arboreal	-0.83	0.81	-0.21	-0.62
Hyaenidae		Terrestrial	-0.12	0.07	1.25	-1.31
Herpestidae		Terrestrial	-1.48	-0.47	-1.65	-0.31
Nandiniidae		Arboreal	0.55	1.11	-0.57	-1.00
Herpestidae		Terrestrial	-0.53	1.26	0.59	-0.19
Eupleridae		Arboreal & Terrestrial	2.76	1.56	-1.98	-1.00
Canidae		Rock Climbing	-0.58	-1.26	2.78	-1.35
Moschidae		Terrestrial & Rock climbing	1.18	0.48	4.32	0.17
Tayassuidae		Terrestrial	1.03	-3.19	0.58	0.25
Equidae		Terrestrial	2.95	-5.73	0.42	0.75
Cervidae		Terrestrial	-0.57	-2.15	1.13	0.31
Giraffidae		Terrestrial	2.34	-4.20	0.07	0.56
Bovidae		Terrestrial	0.24	-2.51	0.81	0.31
Bovidae		Terrestrial & Rock climbing	2.13	-3.98	0.10	0.61
Bovidae		Terrestrial	0.92	-3.04	0.55	0.43
Bovidae		Terrestrial	1.61	-3.46	0.35	0.25
Bovidae		Rock Climbing	0.63	-2.81	0.65	0.38
Bovidae		Rock Climbing	1.60	-3.48	0.28	0.62
Bovidae		Rock Climbing	1.33	-3.23	0.36	0.51
Bovidae		Terrestrial & Rock Climbing	2.45	-4.37	0.04	0.66
Bovidae		Terrestrial	4.35	-5.80	-0.75	1.18
Bovidae		Rock Climbing	2.79	-4.64	-0.05	0.58
Bovidae		Terrestrial & Rock climbing	5.47	-7.24	-1.03	1.10
Bovidae		Terrestrial	5.45	-7.17	-0.89	0.80
Bovidae		Terrestrial	2.88	-4.62	-0.10	0.59
Sciuridae		Arboreal	-2.21	0.15	0.26	-0.75
Sciuridae		Arboreal	-1.56	0.05	-0.63	0.02
Sciuridae	1998.117.16	Arboreal & Terrestrial	-3.42	0.08	-0.60	-0.99
Sciuridae	1960.273.1	Arboreal & Terrestrial	-3.50	0.28	0.19	-0.49
Sciuridae	1984.144.18	Digging & Terrestrial	-0.84	0.46	-0.05	-0.33
Sciuridae		Arboreal	-3.15	-0.12	-0.70	0.23
Sciuridae		Arboreal	-2.82	0.28	-0.38	-0.22
Sciuridae		Arboreal	-2.79	0.35	-1.29	0.53
Sciuridae		Arboreal	-2.93	0.09	0.00	-0.10
Sciuridae		Arboreal	-3.16	0.20	-0.60	0.09
Sciuridae		Arboreal	-3.08	0.09	0.42	-0.08
Sciuridae		Arboreal	-2.58	0.27	-1.47	0.14
Sciuridae		Arboreal	-2.68	0.59	-0.73	0.16
Sciuridae		Digging & Terrestrial	-3.03	0.25	0.98	-0.30
Sciuridae	A5.1.1901	Arboreal	-0.96	0.32	-0.52	-0.40
Sciuridae	4.8.55.44	Arboreal	-1.90	-0.12	-1.57	-0.82

Sciuridae	1981-2497	Arboreal	-2.25	0.38	-0.16	0.02
Sciuridae	A9.8.98.21	Arboreal	-2.23	0.46	-0.56	0.13
Sciuridae	A31.12.14.9.1	Arboreal	-3.40	0.02	-0.09	0.01
Sciuridae	D.312a	Digging & Terrestrial	-2.80	0.36	0.33	-0.43
Sciuridae	1981.5	Digging & Terrestrial	-2.16	-0.05	-0.90	-0.33
Sciuridae	D.379e	Arboreal	-2.25	0.48	-0.20	0.48
Sciuridae	D.350e	Arboreal	-2.82	0.33	1.09	-0.21
Geomyidae	A9.8.98.16	Digging	-3.33	-0.10	1.15	-0.11
Geomyidae	D.301	Digging	-2.67	-0.33	2.04	0.00
Macropodidae	A.28.11.1900	Arboreal	1.17	0.17	-0.99	0.08
Macropodidae	D223	Terrestrial	1.61	0.80	-1.02	0.93
Phalangeridae	D197c	Arboreal	-0.47	0.64	0.53	-0.17
Pseudocheiridae	D202	Arboreal	-1.27	0.92	1.49	-0.20
Phalangeridae	29.7.80.15	Arboreal	-2.31	-0.07	-0.38	-0.74
Thylacomyidae	D259a	Digging & Terrestrial	-1.05	-0.84	0.45	2.93
Didelphidae	14.12.97.7	Arboreal & Terrestrial	-3.43	0.75	-0.18	0.51
Dasyuridae	1981.35	Terrestrial	-3.94	-0.45	-0.88	-0.83
Didelphidae	4.2.1900	Arboreal & Terrestrial	-3.06	0.97	-1.66	1.18
Dasyuridae	D257	Terrestrial	-2.49	0.13	0.87	0.39
Didelphidae	A14.2.26.1	Arboreal & Terrestrial	-1.19	0.35	-1.42	0.46
Tachyglossidae	17.2.79	Terrestrial	-1.58	0.21	-0.15	-0.47
Ornithorhynchidae	1981-7	Semi-aquatic	1.94	0.28	-3.10	-0.80
Dasypodidae		Terrestrial	-1.49	0.12	0.11	0.56
Cyclopedidae	12.7.58	Arboreal & Terrestrial	-1.23	-1.79	0.09	0.02
Myrmecophagidae	18.11.69.12	Arboreal & Terrestrial	-0.89	-0.26	-0.85	-0.23
Phalangeridae	D197c	Arboreal	-0.76	0.46	-0.01	0.19
Macropodidae	D216	Terrestrial	-0.88	-0.22	-0.68	0.87
Macropodidae	D214c	Terrestrial	-0.35	0.06	1.20	-0.18
Phascolarctidae		Arboreal	-1.35	0.78	0.62	0.11
Myrmecophagidae	D243	Terrestrial	1.05	-1.31	-1.76	-1.32
Manidae	D578	Terrestrial	-0.78	0.11	-3.03	-0.66
Chlamyphoridae	1984.144.12	Digging & Terrestrial	-1.46	0.40	1.37	0.10
Choloepodidae	2.11.60.1	Arboreal	0.30	-1.43	-0.98	0.08
Muridae	1981-205	Terrestrial	-4.06	1.11	0.36	-0.58
Ctenodactylidae	D49	Rock Climbing	-3.31	-0.52	-2.67	-0.35
Muridae	1982.944	Arboreal & Terrestrial	-3.20	0.33	-0.81	-0.93
Soricidae	1981-1632	Arboreal & Terrestrial	-3.80	0.19	1.20	-0.26
Tupaiidae	A21.12.01.4	Terrestrial	-3.11	-0.15	-2.55	0.02
Tenrecidae	1980.773.3	Terrestrial & Rock climbing	-2.12	0.57	0.88	2.28
Cricetidae	D354b	Terrestrial	-3.71	0.00	-0.90	-0.07
Petauridae	A.6.7.1900.2	Arboreal	-3.15	0.46	1.91	-0.27
Dasyuridae	D274a	Terrestrial	-3.81	0.58	-0.77	-0.41
Ochotonidae		Terrestrial & Rock climbing	-2.86	-0.72	-1.17	-0.27
Hominidae	12.10.67	Arboreal	2.45	1.24	0.23	-0.07
Sciuridae	16.2.78.11	Digging & Terrestrial	-2.38	0.23	-0.45	-0.23
Sciuridae		Digging & Terrestrial	-2.05	0.13	-0.53	0.13
Sciuridae	D306a	Arboreal	-2.83	0.02	0.21	-0.37

Macropodidae		Terrestrial	0.93	-0.45	-0.22	0.36
Geomyoidea	D448a	Digging	-2.29	-0.58	-0.16	-0.01
Sciuridae	D314	Digging & Terrestrial	-2.41	0.33	-0.79	-0.20
Sciuridae	D3456	Arboreal & Digging	-3.25	-0.26	1.03	0.10
Sciuridae	1982.791	Arboreal	-3.27	0.23	-0.95	0.19
Sciuridae	1984.144.17	Arboreal & Digging	-2.23	0.09	-0.31	-0.03
Geomyoidea	D447a	Digging	-3.30	-0.52	1.80	0.27
Sciuridae	D309a	Digging & Terrestrial	-3.31	0.36	-1.01	0.08
Sciuridae	14.12.97.70	Arboreal & Terrestrial	-3.51	0.49	0.87	0.06
Heteromyidae	A9.8.98	Digging	-3.15	-0.54	0.15	-0.31
Lorisidae	139	Arboreal	-1.74	0.28	2.22	1.72
