# Supplementary material for: Investigating Foot Morphology in Rock Climbing Mammals: Inspiration for Biomimetic Climbing Shoes
Source: Biomimetics (Basel). 2022 Dec 24;8(1):8. doi: 10.3390/biomimetics8010008 (PMC9844278; doi:10.3390/biomimetics8010008)
Supplement: Supplementary file 1 [file biomimetics-08-00008-s001.zip › SupplementaryMethods.pdf]

Investigating foot morphology in rock climbing mammals: inspiration for biomimetic climbing shoes

Section 1

Figure S1 shows the measurements used to obtain the *Quantitative Metrics* (Table 1 of the manuscript) of the feet from photographs. Hoovs were assumed to be more functionally similar to a palmer surface. As such, we treated hooves as anterior pads and allocated zero values to the digit and posterior pad metrics for both the front and rear feet.

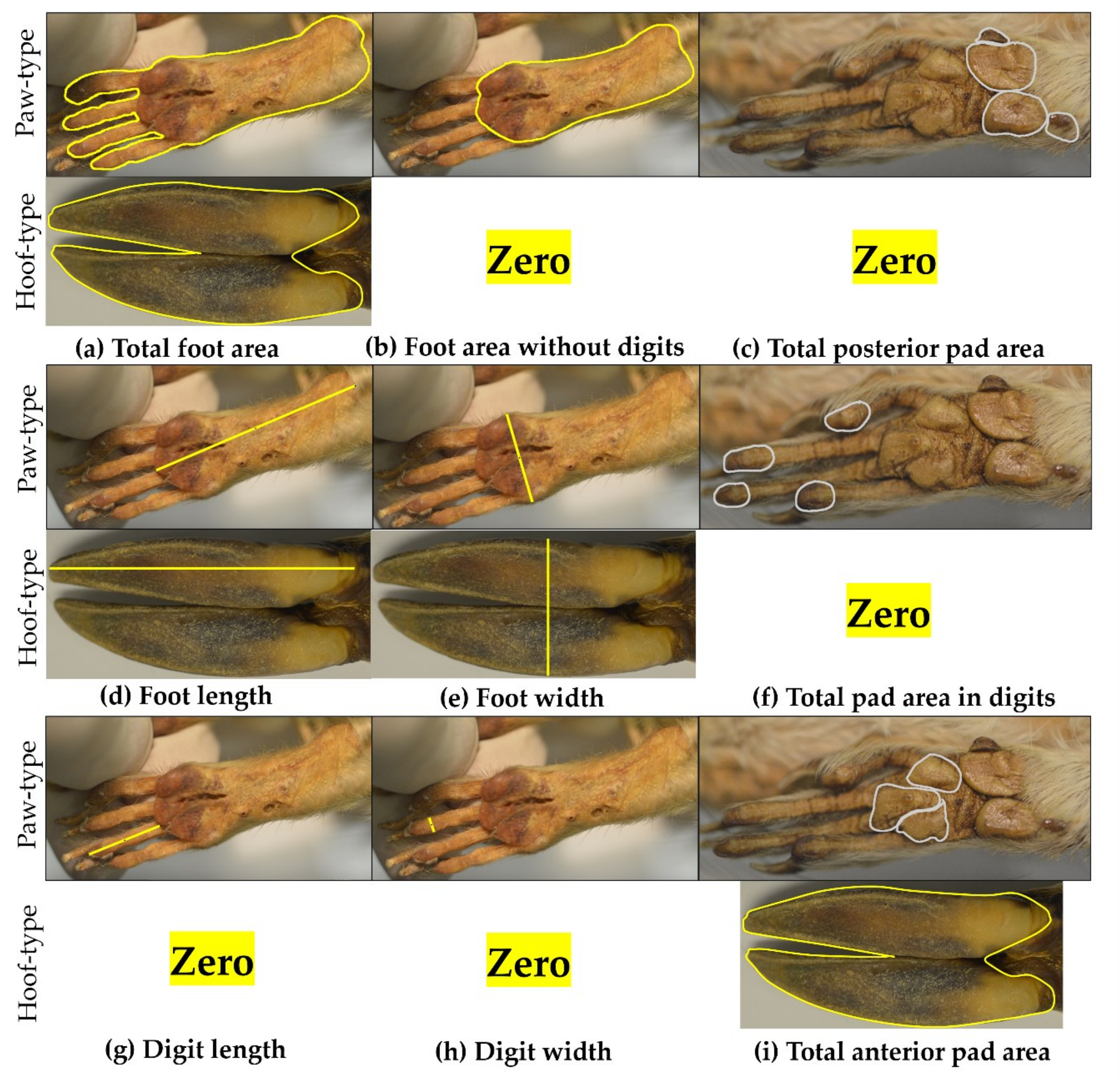

Figure S1. Summary of measurements from photographs for a paw-type foot and a hoof-type foot, including: area of the foot with the digits (excluding the claws) (**a**) and without the digits (**b**), Total Posterior pad area (**c**), Foot length (**d**), Foot width (**e**), Total pad area on digit (**f**), Length from the tip of the digit to the base (excluding claws), (**g**), Width of widest point of the digit (**h**), Total anterior pad area (**i**).

Figure S2 shows different pad textures examples. Circular textures were easy to identify (Figure S2a). However, in relatively smooth textures, these could range from horizontal grooves (Figure S2b), irregular (Figure S2c) and very smooth (Figure S2d). These textures varied across a continuum and could not really be objectively seperated in a repeatable manner, therefore, these were all grouped under the "smooth-ish" category.

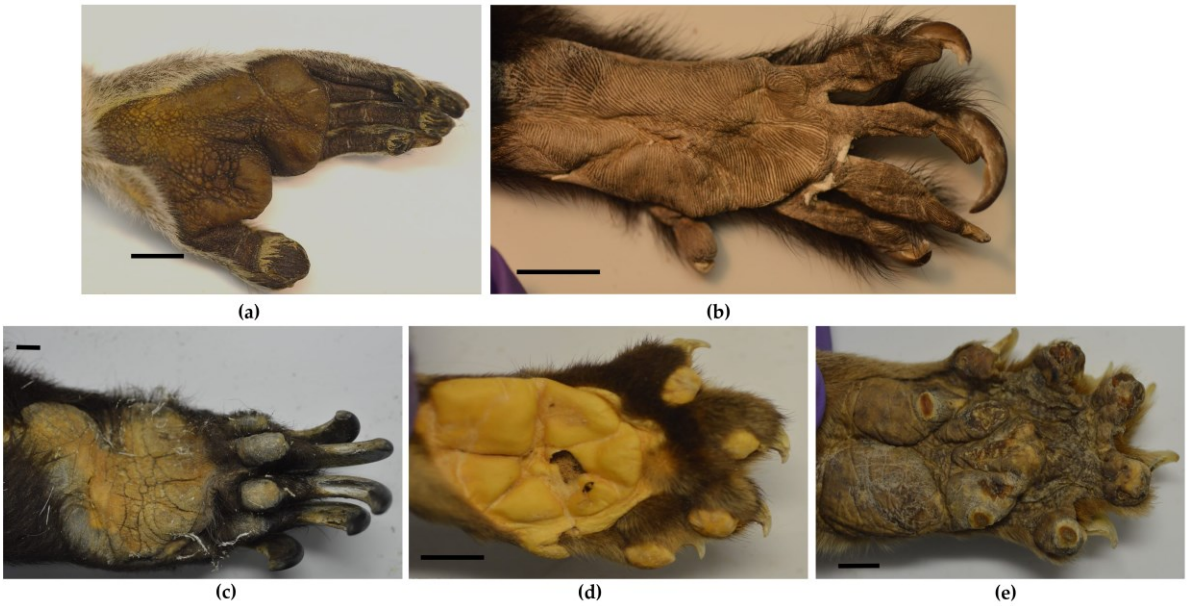

Figure S2. Summary of pad textures for (**a**) Mongoose lemur (*Eulemur Mongoz*), circular textures; (**b**) Goeldi's monkey (*Callimico goeldii*), smooth-ish; (**c**) Sun bear (*Helarctos malayanus*), smooth-ish; (**d**) African palm civet (*Nandinia Binotata*), smooth-ish; (**e**) Fossa (*Cryptoprocta ferox*), smooth-ish. Scale bar represents 10 mm.

Section 2

Figure S3 shows a phylogenetic tree of all species used in this study (n=166), based on a mean consensus tree of 1000 trees from Vertlife.org (downloaded 06/10/22), based on Upham et al. [41]:

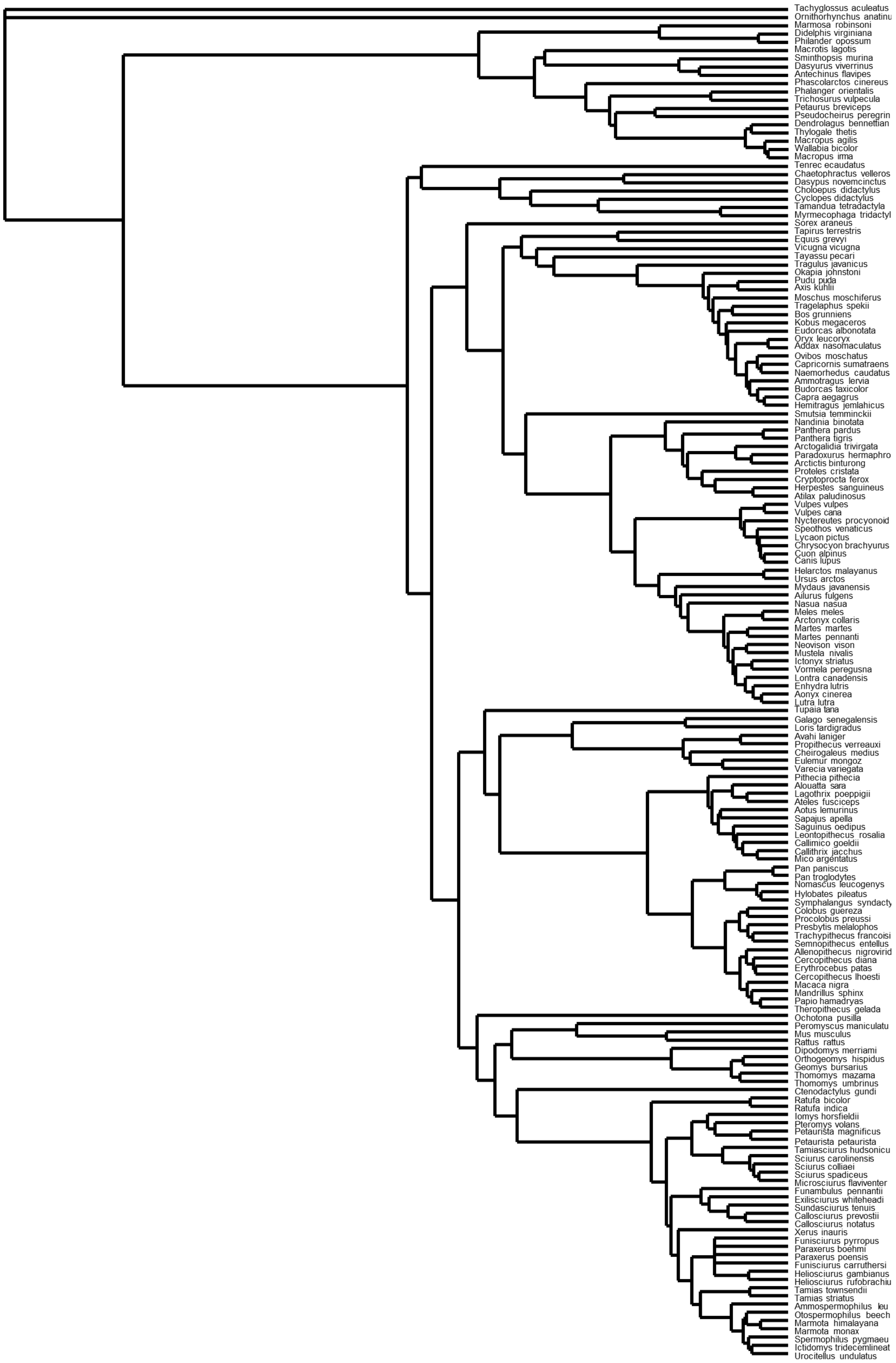

Figure S3. Phylogenetic consensus tree used for Phylogenetic ANOVA statistics
